# The Oxford Visual Perception Screen: Development and normative data of a standardised assessment for visual perception difficulties

**DOI:** 10.1177/02692155251315606

**Published:** 2025-02-27

**Authors:** Kathleen Vancleef, Ruby Castellani, Rebecca Shorthose, Catherine Guo, Merak Fulin Cai, Federica Guazzo, Nele Demeyere

**Affiliations:** 1 Department of Experimental Psychology, 6396 University of Oxford, Oxford, UK; 2 Department of Psychology, 3057 Durham University, Durham, UK; 3 Nuffield Department of Clinical Neuroscience, 6396 University of Oxford, Oxford, UK

**Keywords:** Perception, visual impairment, cognitive impairment, assessment, normative data

## Abstract

**Objective:**

We aimed to develop and standardise a practical systematic screening tool for visual perception impairments after a stroke to replace current subjective methods.

**Design:**

A mixed methods study including a cross-sectional study and a case series.

**Setting:**

In the community and on stroke rehabilitation units.

**Participants:**

Older volunteers without a neurological history contributed to normative data. Patients with ocular conditions or a stroke took part in our case series.

**Instrument:**

The Oxford Visual Perception Screen.

**Main measures:**

For each task of the Oxford Visual Perception Screen, we determined 5^th^ centile cut-off scores. We further explored effects of age, visual acuity and gender on visual perception through generalised linear models.

**Results:**

Oxford Visual Perception Screen is a 15-min paper-and-pen assessment comprising 10 tasks including picture naming, star counting and reading. Normative data of 107 participants demonstrated persistent high performance with most cut-offs near ceiling. Apart from the Figure Copy (*Z* = 6.57, *p* < 0.001) and Global Shape Perception task (*Z* = 3.32, *p* < 0.001) we found no evidence for an effect of visual acuity on OxVPS. An effect of age was only observed in the Face Recognition (*Z* = 3.61, *p* < 0.001) and Reading task (*Z* = −3.50, *p* < 0.001). No effects of gender were observed. A series of eight cases illustrates the interpretation of OxVPS.

**Conclusion:**

We present the Oxford Visual Perception Screen, a standardised visual perception screening tool alongside normative data and illustrative cases. The Oxford Visual Perception Screen can potentially change screening for visual perception impairments in clinical practice and is available at https://oxvps.webspace.durham.ac.uk/.

## Introduction

Visual perception is the dynamic process of perceiving the environment through sensory inputs and translating the sensory input into meaningful concepts.^1^ Although visual perceptual problems are highly common,^2^ assessment of visual perception problems after stroke is challenging. Existing visual perception test batteries take at least 45 min to complete^3,4^ making them unsuitable for systematically screening all stroke survivors in time- and resource-poor acute settings,^5^ and who themselves often present with fatigue^6^ and impaired sustained attention.^7^ In addition, current instruments require considerable training in administration and interpretation,^3,8^ making them less accessible to healthcare professionals. Therefore, healthcare professionals typically rely on patients' self-reports and observations.^9,10^ Because the sensitivity of any screening tool is significantly lowered when patients are unable to report their symptoms, many impairments may currently be missed.^11^

The Oxford Visual Perception Screen was developed to meet the need for a quick, easy to administer, standardised assessment that is suitable for stroke survivors. To support content and face validity, the selection of which visual perceptual impairments to screen for was guided by expert consensus based on a Delphi study^12^ and further informed by a recent survey with healthcare professionals.^9^ The selection of tasks to be included was guided by psychometric properties and by health professionals’ opinions (to maximise adoption) and practicality of tasks. The format and layout of the Oxford Visual Perception Screen was inspired by the successful Oxford Cognitive Screen,^13^ which also maximises the accessibility of the tasks for stroke survivors by being inclusive for those with unilateral weakness affecting their hand, communication difficulties, visual neglect, and fatigue.

Iterative drafts of the Oxford Visual Perception Screen were reviewed by internationally recognised experts in neuropsychology research, health professionals, healthy volunteers, and stroke survivors. Over 30 iterations of improvements resulted in the current version of the Oxford Visual Perception Screen, designed to be a practical and acceptable screening tool. The Oxford Visual Perception Screen is available to download on https://oxvps.webspace.durham.ac.uk/.

Here we report a normative study to establish cut-off scores for normal performance on OxVPS and illustrate the interpretation of OxVPS through a case series.

## Methods

Participants were included in the normative study if they were neurologically healthy, adult and English-speaking. Exclusion criteria for all participants were psychiatric conditions affecting their daily life and a history of neurological conditions with potential long-lasting effects. People who reported mild depression or anxiety controlled by medication, who experienced a transient ischaemic attack or headaches were not excluded.

For our case series, we included stroke survivors who had demonstrated visual perceptual difficulties in the Rivermead Perceptual Assessment Battery.^14^ In addition, we included participants without a stroke but with self-reported ocular conditions. Exclusion criteria were the same as above.

Participants were recruited through social media (Facebook and Twitter), our research group's participant pool of healthy older volunteers, care homes, through social groups for people of an older age, and at stroke rehabilitation units.

All participants provided written informed consent. All procedures were reviewed by the Psychology Ethics Committee at Durham University or the Health Research Authority Derby Research Ethics Committee and were given a favourable opinion (reference numbers PSYCH-2022-01-19T13_53_52 and 23/EM/0086).

Following Crawford's recommendations, we established that a normative sample of 100 participants would enable us to calculate the 5^th^ percentile with a 95% confidence interval ranging from the 2^nd^ to 8^th^ percentile.^15^

Data were collected between May 2023 and January 2024 by student research assistants in the participant's location of choice such as their home, their room in a care home, in a stroke rehabilitation unit, community halls, or at a research lab at Durham University. All locations were in County Durham, Surrey, and North Yorkshire, countries in England in the United Kingdom of Great Britain and Northern Ireland.

The Oxford Visual Perception Screen is a 15-min screening tool in paper format which briefly assesses visual perception including object recognition, face recognition, reading, eye-hand coordination (visuo-constructive skills) as well as visuospatial neglect, and more. Across ten disparate tasks, patients are asked to recognise objects, faces, read a short paragraph, select targets, and draw a geometrical figure. Except for the drawing, cancellation, and reading task, all tasks are multiple choice (overcoming expressive communication issues), images are presented vertically (avoiding confounds due to visual neglect), and patients can respond with pointing gestures with their unaffected hand (in case of any upper-limb weakness). The test results indicate which visual perceptual problems are likely present in a patient. A total score indicates the extent of the visual perceptual problems.

An overview of the ten tasks of the Oxford Visual Perception Screen and impairments is given in Table 1. Further details are available in the Oxford Visual Perception Screen manual.^16^ The Oxford Visual Perception Screen is freely available to download for non-commercial use at https://oxvps.webspace.durham.ac.uk/.

**Table 1.** Description of tasks of the Oxford Visual Perception Screen (version 2.1).

Task name	Task description	Impairments screened for
Self-Evaluation	This task records subjective visual complaints through three questions on whether the patient noticed any difficulties with perception in general, perception of motion, and perception of colours.	Blindsight: patients would answer they cannot see anything but their performance in other tasks of the Oxford Visual Perception Screen is above chance Anton-Babinsky syndrome: patients would deny having any difficulties while performance in other tasks of the Oxford Visual Perception Screen is lower than the cut-off for normal visual perception Achromatopsia Akinetopsia
Picture Naming	In this task, the patient is shown a black and white line drawing (e.g. bear) at the top of the page and is asked what it is a picture of. Five possible answers are given. One of the incorrect options is semantically related to the drawing but not visually (e.g. kangaroo), another is visually related but not semantically (e.g. table), another is visually and semantically related (e.g. dog), and the last one is unrelated (e.g. car). Four drawings are presented at different pages.	Optic aphasia Associative agnosia: patients would also have difficulties with Semantic Info Apperceptive agnosia: patients would also have difficulties with Semantic Info, Global Shape Perception Cortical blindness: patients would also have difficulties with any other task
Semantic Info	The patient is shown four black and white line drawings of objects alongside five words and is asked which word goes best with each image. The words are all associated with each other but only one is strongly associated with the image. For instance, a drawing of a rabbit will be shown alongside the words ‘carrot’, ‘pear’, ‘onion’, ‘tomato’, ‘potato’. All these are fruit or vegetables, but only one, ‘carrot’, is strongly associated with a rabbit.	Associative agnosia: patients would also have difficulties with Picture Naming Apperceptive agnosia: patients would also have difficulties with Picture Naming, Global Shape Perception Cortical blindness: patients would also have difficulties with any other task
Global Shape Perception	In this task, a fragmented outline of an irregular shape is shown at the top of each of the four pages of this task. Underneath, four other fragmented shapes are presented. Patients need to pick the shape that is most similar to the target shape at the top of the page. To ease the distinction between the target shape and the options to choose from, the target shape is made up of thicker line fragments.	Apperceptive agnosia: patients would also have difficulties with Picture Naming, Semantic Info Cortical blindness: patients would also have difficulties with any other task Simultanagnosia: patients would also have difficulties with Item Counting
Item Counting	In this task, patients are asked to count the number of stars presented on each of the four pages. Four options are given underneath each stimulus. Besides the correct number, the options include numbers in proximity to the correct number with at least one number smaller than the correct number.	Cortical blindness: patients would also have difficulties with any other task Simultanagnosia: patients would also have difficulties with Global Shape Perception
Simple Feature Perception	The patient is shown four straight lines and for each of them is asked if the line is tilted.	Cortical blindness: patients would also have difficulties with any other task
Face Recognition	In each of the four trials of this task, patients are shown five photographs of faces. One happy face at the top of the page and four neutral faces below. They are asked which of the four neutral face photographs is of the same person as the happy face photograph. All faces show a frontal view, have a white background, and cover a similar area in the image. All models wear a neutral black t-shirt and accessories such as jewellery were removed.	Prosopagnosia
Reading	In the reading task, the patient is asked to read a short paragraph. The paragraph consists of exactly 60 words including low frequency words (doughty, snappily), compound words (firefighters, overgrown, sunset, farmhouse, overnight), and a combination of low frequency and compound words (hitchhiker, woodshed, lean-to) evenly spread across the paragraph. The task is timed and any incorrect or omitted words are marked. The reading speed is calculated as the number of correctly read words per minute. An accuracy score is calculated for the low frequency and compound words.	Alexia Neglect dyslexia
Cancellation	In this task, the patient is presented with small heart shapes scattered over a page^a^. Some hearts are complete, others have a gap on the left or right side. They are asked to mark off the complete hearts. The page has to be presented at the body midline of the patient and cannot be moved (unless the patient moves their body midline).	Space-based or egocentric neglect Object-based or allocentric neglect
Figure Copy	Patients are asked to copy a complex geometric figure on a page. The figure shows a rectangle, divided in two halves, with smaller elements like star, circle or triangle placed on specific positions in the two halves^b^. These is no time limit to this task.	Visuo-constructive deficit Global attention deficit Simultanagnosia Apperceptive agnosia

a The task is adapted from the Oxford Cognitive Screen cancellation task,^13^ by reducing the number of hearts targets (30 instead of 50), but keeps the same density of distribution by compressing the vertical search space on the page.

b The figure is identical to the one in the Oxford Cognitive Screen - Plus.^31^

Participants were instructed to wear their habitual correction for a viewing distance of 30–40 cm. We used version 2.1 of the Oxford Visual Perception Screen. Participants could take as much time as needed for each task. They were encouraged to guess on multiple choice questions and were allowed to correct their answers. For each task of the Oxford Visual Perception Screen, a score was calculated as described in the manual.

Acuity was assessed with the LogMar Double Sided Near Vision Card/EDTRS chart a standardised assessment for near visual acuity.^17^ Acuity was defined as the print size for which at least 50% of the letters can be read correctly.^18^

Demographic information and medical information to check exclusion criteria and to describe the sample was requested through a short health questionnaire.

Descriptive statistics on demographic variables were calculated to characterise the sample. The performance on each task of the Oxford Visual Perception Screen was summarised by the median, interquartile range, 5^th^, and 10^th^ centile. Sensitivity analyses were performed to investigate the effects of age, gender, and visual acuity on scores through Generalised Linear Models. For tasks with a negatively skewed distribution of scores (i.e. all scores but reading speed and asymmetry scores), the observed scores were transformed by subtracting them from the perfect score on the task and modelled with a Poisson link function in a Generalised Linear Model. Scores on other tasks were modelled with an Identity link function. A Bonferroni correction for multiple comparisons was used and the significance level was adjusted to 0.0015 to reflect a family-wise error rate of 0.05. Missing data were not replaced.

## Results

**Figure 1.**
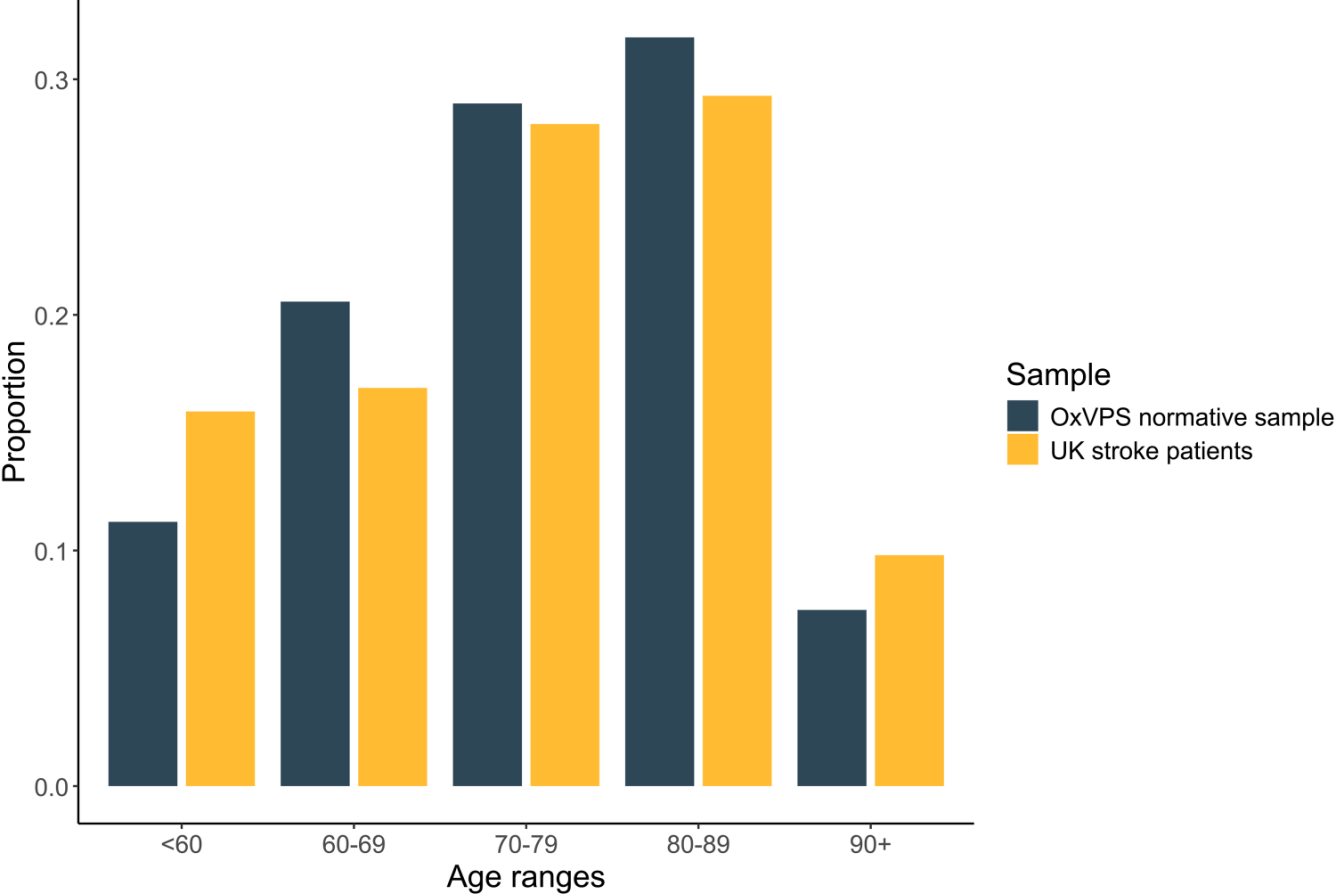
Distribution of age of the participants in our normative sample (in dark, *N* = 107) compared to the distribution of age of stroke patients admitted to hospital in England, Wales and Northern Ireland between April 2022 and March 2023 (in light, *N* = 91,162; source:^19^).

All 108 normative group participants completed the Oxford Visual Perception Screen and a near visual acuity test; one participant was excluded because of a neurological condition. Data of all remaining 107 participants was included and analysed. The median duration of a session was 20 min with an interquartile range from 16 to 27 min. The distribution of ages in our normative sample is similar to the distribution of ages in stroke patients^19^ as can be viewed in Figure 1. Further details of all 107 participants are reported in Table 2.

**Table 2.** Demographic details of participants in our normative sample.

	Mean	SD	Count^a^	Percent
Age (years)	74.21	11.24		
Gender				
Man			36	34
Woman			71	66
Ethnic origin				
White			105	98
Unknown			2	2
Education				
No qualifications			3	3
Below secondary education			7	7
Secondary education			5	5
Upper secondary and advanced further education			9	8
Higher education			81	76
Unknown			2	2
Handedness				
Ambidextrous			3	3
Left-handed			8	7
Right-handed			96	90
Visual acuity (logMAR)	0.12	0.19		
Glasses				
None			12	11
Distance glasses			14	13
Reading glasses			40	37
Varifocal or bifocal glasses			49	46
Eye conditions				
None			64	60
Cataracts			23	21
Dry eye			3	3
Glaucoma			5	5
Macular Degeneration			5	5
Unsure			4	4
Other			9	8

a The count data in each category of glasses and eye conditions do not add up to 107 because some participants worn multiple type of glasses (e.g. reading and distance glasses) or had multiple eye conditions.

**Figure 2.**
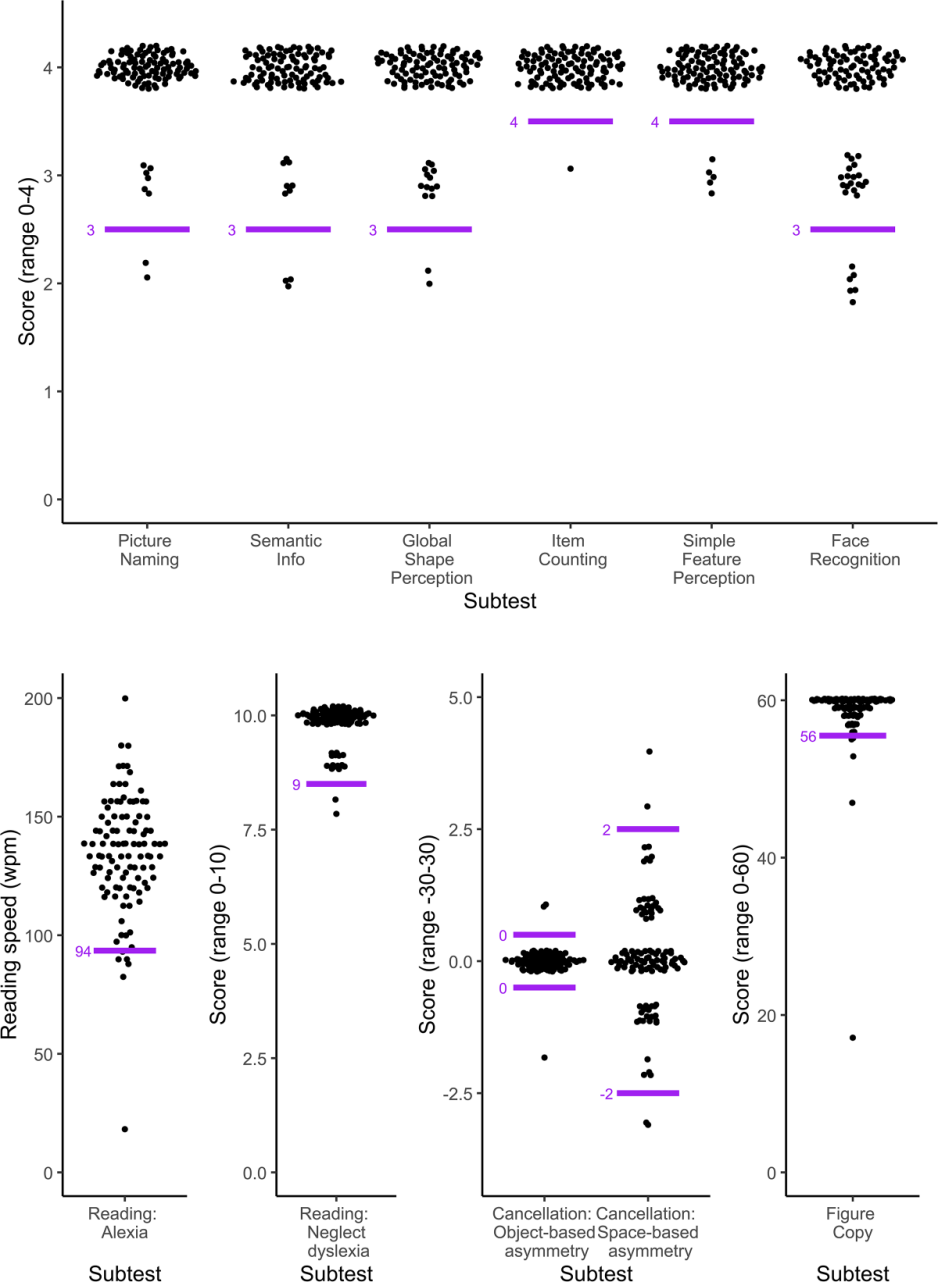
Distribution of scores for each task of the Oxford Visual Perception Screen (version 2.1). The top graph shows the scores of all tasks with a range of scores between 0 and 4. The bottom row of graphs shows the Reading speed in the Reading task in words per minute (no minimum or maximum), the number of correctly read complex words in the Reading task (maximum 10), the Object- and Space-based asymmetry scores (minimum possible score of −30 and maximum of 30) and the score on the Figure Copy task (maximum 60). A small amount of vertical jitter was added to the scores to show overlapping data points and better reflect the distribution of the scores. For instance, the dots around the number 4 in the Picture Naming task all represent participants with a score of exactly 4 out of 4 on the task. The horizontal spread of the dots is proportionate to the number of data points at that value. The short horizontal lines indicate the cut-off scores for each task of the Oxford Visual Perception Screen (version 2.1). For clarity, these lines were placed on the division line between ‘impaired’ and ‘unimpaired’ scores rather than at the location of the cut-off value. For instance, the cut-off value for the Picture Naming task is 3, but the short horizontal line is places slightly lower to show that a score of 3 is still within the normal range, while a score of 2 is only observed in less than 5% of our normative sample and therefore indicative for an impairment.

The distributions of the scores for each task of the Oxford Visual Perception Screen were highly skewed (see Figure 2). In Table 3, we report the median, interquartile range and the 5^th^ and 10^th^ centile scores for each task. Two participants who had a visual acuity of only 0.8 logMAR were not able to complete the reading task, but completed all other tasks. For most tasks, the 5^th^ centile score can be used as cut-off score when screening for visual perception impairments and can be taken as indicative for impairment (see last column in Table 3). The exceptions are the Self-evaluation task and the Strategy score of the Figure Copy task which are evaluated qualitatively.

**Table 3.** Descriptive statistics for each task of the Oxford Visual Perception Screen (version 2.1).

Outcome measure per task	*N* ^1^	Median	IQR	5^th^ centile	10^th^ centile	Indicative for ‘impairment’
Self-Evaluation						
Subjective complaints						‘Yes’
Akinetopsia						‘Yes’
Achromatopsia						‘Yes’
Picture Naming	107	4	4–4	3	4	Lower than 3
Semantic Info	107	4	4–4	3	4	Lower than 3
Global Shape Perception	107	4	4–4	3	3	Lower than 3
Item Counting	107	4	4–4	4	4	Lower than 4
Simple Feature Perception	107	4	4–4	4	4	Lower than 4
Face Recognition	107	4	4–4	2.3	3	Lower than 3
Reading						
Neglect dyslexia	105	10	10–10	9	9	Lower than 9
Alexia (wpm)	105	138.5	122.1–150	93.5	103	Lower than 94
Cancellation						
Object asymmetry	107	0	0–0	0 and 0	0 and 0	Lower than 0 or higher than 0
Space asymmetry	107	0	0–0	−2 and 2	−1.7 and 2	Lower than −2 or higher than 2
Figure Copy						
Total	107	60	59–60	56	57	Lower than 56
Strategy	51					Not drawing big rectangle first

a Not all outcome measures are available for each participant. Two participants could not complete the reading task due to low visual acuity. The Figure Copy strategy was only recorded in 51 participants due to an administrative error.

The estimated coefficients of each Generalised Linear Model, Z statistics and *p*-values are reported in Table 4.

**Table 4.** Effect of age, gender and visual acuity on the Oxford Visual Perception Screen (version 2.1) performance.

	Coefficient	*Z*	*p*
Picture Naming			
Age	0.1518	2.5843	0.0098
Gender	0.1373	0.1882	0.8507
Visual acuity	1.6894	1.006	0.3144
Semantic Info			
Age	0.033	1.0216	0.307
Gender	−1.755	−2.6516	0.008
Visual acuity	2.309	2.1668	0.0302
Global Shape Perception			
Age	0.007	0.3016	0.7629
Gender	−0.328	−0.6386	0.5231
Visual acuity	3.102	3.3215	0.0009*
Item Counting			
Age	0.2294	0.7416	0.4584
Gender	−19.323	−0.0027	0.9979
Visual acuity	−7.5032	−0.8299	0.4066
Simple Feature Perception			
Age	0.0448	0.8868	0.3752
Gender	−0.8603	−0.9282	0.3533
Visual acuity	3.2349	1.9398	0.0524
Face Recognition			
Age	0.0945	3.605	0.0003*
Gender	−0.7307	−1.9722	0.0486
Visual acuity	1.2644	1.5236	0.1276
Reading: Alexia (wpm)			
Age	−0.6921	−3.5019	0.0007*
Gender	2.0869	0.4575	0.6483
Visual acuity	−36.7893	−2.8486	0.0053
Reading: Neglect dyslexia			
Age	−0.0141	−0.6954	0.4868
Gender	0.051	0.1022	0.9186
Visual acuity	1.0431	0.9561	0.339
Cancellation: Object asymmetry			
Age	0.0172	1.822	0.0714
Gender	−0.1166	−0.5392	0.5909
Visual acuity	−0.0256	−0.0474	0.9623
Cancellation: Space asymmetry			
Age	−0.0032	−1.4771	0.1427
Gender	−0.0511	−1.0412	0.3002
Visual acuity	−0.0038	−0.0309	0.9754
Figure Copy: Total			
Age	0.0241	3.1046	0.0019
Gender	0.2535	1.4083	0.159
Visual acuity	2.238	6.5682	<0.0001*

*p-values below 0.0015.

The observed decrease in performance with increasing age provided supporting evidence that in the population there is an effect of age on performance in the Face Recognition task (*Z* = 3.61, *n* = 107, *p* = 0.0003) and on reading speed in the Reading task (*Z* = −3.50, *n* = 105, *p* = 0.0007). In addition, we observed evidence that visual acuity negatively affects performance in the Global Shape Perception (*Z* = 3.32, *n* = 107, *p* = 0.0009) and the Figure Copy task (*Z* = 6.57, *n* = 107, *p* < 0.0001). Although it has been repeatedly demonstrated that visual acuity decreases with age,^20,21^ the correlation between visual acuity and age in our sample was low (*r* = 0.25), likely because we measured visual acuity while participants were wearing their habitual correction.

In addition to the normative data, we present data on eight example patient cases (see Table 5). Patients with Macular Degeneration that severely impacted their daily life (Case 1–2) failed several tasks of the Oxford Visual Perception Screen but their profile of scores is not indicative of any visual perceptual impairment that the Oxford Visual Perception Screen screens for and can therefore not be mistaken for a visual perception issue. Patients with cataract, glaucoma or dry eye with no to little reduction in near visual acuity and only a mild effect on daily life (Case 3–5) performed well on all tasks of the Oxford Visual Perception Screen. Cases 6–8 were diagnosed with visual perceptual difficulties following an extensive neuropsychological assessment with the Rivermead Perceptual Assessment Battery. Case 6 failed across all tasks of the Oxford Visual Perception Screen and had no subjective complaints about their vision. This profile is indicative of severe cortical blindness. However, their performance in the cancellation task was better than would be expected from a blind patient. Case 7’s scoring profile showed signs of prosopagnosia by failing the Face Recognition task, a visuo-constructive deficit as indicated by a low score on Figure Copy task, alexia, and neglect dyslexia (low score on both measures in the Reading task). They reported that naming colours was difficult and when objects are moving fast ‘their brain can’t keep up’, which is indicative of akinetopsia and achromatopsia. Case 8’s scoring profile was indicative of optic aphasia (failure on Picture Naming, but not on Semantic Info, Global Shape Perception, Item Counting, or Simple Feature Perception), and alexia and neglect dyslexia (low score on both measures in the Reading task).

**Table 5.** The Oxford Visual Perception Screen (version 2.1) profile of nine cases with either sensory vision problems or with a stroke.

	Case 1	Case 2	Case 3	Case 4	Case 5	Case 6	Case 7	Case 8
Neurological Condition	None	None	None	None	None	Stroke	Stroke	Stroke
Eye Condition	MD, C	MD	G, C	C	DE	None	C, Blind in left eye	None
Near visual acuity (logMAR)	0.7	0.8	0.0	0.3	0.3	0.4	0.0	0.4
Effect of eye condition on daily life	Severe	Severe	Mild	Mild	Mild	NA	NR	NA
Oxford Visual Perception Screen total score	6	8	10	10	10	1	6	8
Self-evaluation								
Subjective complaints	NA	NA	NA	NA	NA	No	Yes	No
Akinetopsia	NA	NA	NA	NA	NA	No	Yes	No
Achromatopsia	NA	NA	NA	NA	NA	No	Yes	No
Picture Naming	3	4	4	4	4	1	4	2
Semantic Info	2	4	4	4	4	1	3	3
Global Shape Perception	2	4	4	4	3	2	4	4
Item Counting	4	4	4	4	4	0	4	4
Simple Feature Perception	3	4	4	4	4	3	4	4
Face Recognition	3	3	4	3	4	0	2	3
Reading								
Alexia (wpm)	18	UR	157	150	122	50	87	92
Neglect dyslexia	8	UR	10	10	10	2	8	8
Cancellation								
Object asymmetry	0	0	0	0	0	2	0	0
Space asymmetry	1	4	0	0	−2	−1	−1	1
Time (sec)	356	73	68	40	39	265	93	100
Figure Copy								
Total	57	53	60	60	57	28	55	59
Strategy	1	NR	1	1	NR	0	1	1

MD: macular degeneration; C: cataract; G: glaucoma; DE: dry eye; NR: not recorded; NA: not applicable; UR: unable to read.

## Discussion

With the Oxford Visual Perception Screen, stroke survivors can be screened for 15 visual perceptual impairments. Our normative data of 107 neurologically healthy older volunteers provide a standardised benchmark for normal performance on the Oxford Visual Perception Screen. The scores on the tasks were highly skewed with many healthy volunteers obtaining the maximum score on each task. Based on our normative data, we have calculated 5^th^ centile cut-off scores for normal performance on each task. The Oxford Visual Perception Screen was designed as a screening tool. This means it is not designed to comprehensively diagnose visual perception problems. Instead, the results of the Oxford Visual Perception Screen should provide pointers to which perceptual difficulties might be present, in order to support referral for comprehensive assessment and inform interim rehabilitation advice.

The Oxford Visual Perception Screen aims to fill the gap in existing visual perception assessments for a quick and easy to use screening tool that is accessible for stroke survivors. Although some existing tests like the Rivermead Perceptual Assessment Battery^3^ and the Occupational Therapy Adult Perception Screening Test^8^ have excellent psychometric properties,^22–24^ they are not always feasible to complete with stroke survivors at the acute stage because of their length, because of reliance on verbal communication, or because cumbersome testing materials that make it difficult to complete at bedside. These practical barriers mean existing screening tests are not often used in clinical settings.^9^ With the development of the Oxford Visual Perception Screen, we focused on practicality and key requirements as identified by health professionals. The Oxford Visual Perception Screen is a screening test that takes 15 min, making it feasible for stroke survivors with limited concentration in the first few days after a stroke. Health professionals can learn to administer, score and interpret the Oxford Visual Perception Screen by watching a 20-min video. In addition, the Oxford Visual Perception Screen is portable and compact, making it feasible to be completed at bedside. Through these features, the Oxford Visual Perception Screen has the potential to change the screening for visual perception impairments in clinical practice.

A strength of the study is the age range of our normative sample: 45% of our volunteers were above 80 years old and the average age was 74.2 years old (SD = 11.2). A limitation is the education level and ethnicity in our normative sample. Seventy-three percent of our volunteers completed higher education compared to only 28% in the general population of England and Wales.^25^ In addition, nearly all our participants were of white ethnicity. The effect of education and ethnicity on performance in visual perception tasks is debatable^26,27^ with some studies highlighting the need for ethnicity specific normative data for neuropsychological tests.^28,29^ In future, the effect of education and ethnicity on performance in the Oxford Visual Perception Screen should be further explored.

A second limitation are the prerequisites for the Oxford Visual Perception Screen. Patients need to have a good understanding of the language to comprehend the instructions and understand the answer options. In addition, a basic education with literacy is a condition for the Reading task. Furthermore, our normative data showed that a minimum near visual acuity of 0.7 logMAR with habitual correction (equivalent to 6/30 or 20/100 Snellen and 0.20 decimal acuity) is required for the Reading task. The other tasks of the Oxford Visual Perception Screen have not been tested in people with poorer near vision than 0.8 logMAR (equivalent to 6/38 or 20/125 Snellen and 0.16 decimal acuity). Finally, a note should be made about any cognitive (e.g. executive functions), communication (e.g. aphasia) or physical impairments (e.g. arm weakness, fatigue) that might have an influence on performance, and thus for the Oxford Visual Perception Screen to be conducted following first-line screening on these aspects (e.g. with the Oxford Cognitive Screen^13^).

As a third limitation, the current study does not evaluate the diagnostic accuracy, reliability and validity of the Oxford Visual Perception Screen, for which work is ongoing. To evaluate the diagnostic accuracy (e.g. sensitivity, specificity and false positive and false negative rate), a comparison must be made between impairment classifications of patients’ visual perception based on the Oxford Visual Perception Screen and on a gold standard test for visual perception. The current data do not allow such evaluation. This will need to be addressed in future research. Although the design choices and case examples provide some initial evidence of validity this is only preliminary. For instance, the Oxford Visual Perception Screen was designed so common ophthalmological conditions in elderly people^30^ lead to a different pattern of scores and mistakes compared to the visual perceptual impairments. The case series indeed suggest that although patients with severe ocular conditions can fail some of the tasks in the Oxford Visual Perception Screen, their mistakes can usually be explained by their ocular condition and/or their profile of failed tasks does not correspond to any of visual perceptual impairments that the Oxford Visual Perception Screen screens for. In the future, the effect of common sensory vision conditions like cataracts, glaucoma and macular degeneration could be systematically evaluated in a sample for which detailed optometric and ophthalmological information is available. Until then, a note should be made of any ophthalmological or sensory vision problems that might affect the interpretation of the Oxford Visual Perception Screen scores. In addition, research into test-retest reliability and inter-rater reliability will show if the Oxford Visual Perception Screen test scores are stable across test sessions and examiners. A sufficiently powered validation study with stroke survivors assessing convergent and divergent validity is essential to develop the evidence-base to support the use of the Oxford Visual Perception Screen in clinical practice.

In sum, here we presented a new standardised screening tool for visual perception following stroke along with normative data and cut-offs. Following future validation research, the Oxford Visual Perception Screen has the potential to improve the detection of visual perception difficulties after stroke and support the planning of subsequent in-depth assessment and decisions on interim rehabilitation advice until a diagnosis is confirmed. The Oxford Visual Perception Screen is available to download at https://oxvps.webspace.durham.ac.uk/.

**Clinical messages**

- The Oxford Visual Perception Screen provides a standardised assessment to systematically screen nearly all stroke survivors for visual perception difficulties.
- The Oxford Visual Perception Screen screens for 15 different visual perception impairments in 10 short tasks.
- Normative data of 107 healthy older volunteers provide a benchmark for normal performance on the Oxford Visual Perception Screen through cut-off scores for each task.
- The Oxford Visual Perception Screen makes screening for visual perception difficulties more accessible through a quick 15-min assessment that is easy to administer and interpret by health care professionals from various disciplines.

## References

[1] Bouska MJ Kauffman NA Marcus SE . Disorders of the visual perceptual system. In: Umphred DA (eds) Neurological rehabilitation. 2nd edition. St. Louis, MO: The C. V. Mosby Company, 1990, pp.705–740.

[2] Edmans J Lincoln N . The frequency of perceptual deficits after stroke. Clin Rehabil 1987 Nov 1; 1: 273–281.

[3] Whiting S Lincoln NB Bhavnani G , et al. Rivermead Perceptual Assessment Battery. Windsor: NFER-Nelson, 1985. 10.1080/J003v03n03_18 23947471

[4] Warrington EK James M . The visual object and space perception battery: VOSP. Bury St. Edmunds: Thames Valley Test Co, 1991.

[5] Cooke DM McKenna K Fleming J . Development of a standardized occupational therapy screening tool for visual perception in adults. Scand J Occup Ther 2005 Jun 12; 12: 59–71. 16392761 10.1080/11038120410020683-1

[6] Alghamdi I Ariti C Williams A , et al. Prevalence of fatigue after stroke: a systematic review and meta-analysis. Eur Stroke J 2021 Dec 1; 6: 319–332. 35342803 10.1177/23969873211047681 PMC8948505

[7] Varkanitsa M Godecke E Kiran S . How much attention do we pay to attention deficits in poststroke aphasia? Stroke 2023 Jan; 54: 55–66. 36542078 10.1161/STROKEAHA.122.037936 PMC9927868

[8] Cooke D . Function for Life. 2023 [cited 2024 Jan 29]. Occupational Therapy Adult Perceptual Screening Test (OT-APST). Available from: https://www.functionforlife.com.au/

[9] Colwell MJ Demeyere N Vancleef K . Visual perceptual deficit screening in stroke survivors: evaluation of current practice in the United Kingdom and Republic of Ireland. Disabil Rehabil 2022 Oct 23; 44: 6620–6632. 34455876 10.1080/09638288.2021.1970246

[10] Vancleef K Colwell MJ Hewitt O , et al. Current practice and challenges in screening for visual perception deficits after stroke: a qualitative study. Disabil Rehabil 2022 May 8; 44: 2063–2072. 33016779 10.1080/09638288.2020.1824245

[11] Hanna KL Hepworth LR Rowe F . Screening methods for post-stroke visual impairment: a systematic review. Disabil Rehabil 2017 Dec 4; 39: 2531–2543. 27669628 10.1080/09638288.2016.1231846

[12] de Vries SM Heutink J Melis-Dankers BJM , et al. Screening of visual perceptual disorders following acquired brain injury: a Delphi study. Appl Neuropsychol Adult 2018 May 4; 25: 197–209. 28098479 10.1080/23279095.2016.1275636

[13] Demeyere N Riddoch MJ Slavkova ED , et al. The Oxford cognitive screen (OCS): validation of a stroke-specific short cognitive screening tool. Psychol Assess 2015 Sep; 27: 883–894. 25730165 10.1037/pas0000082

[14] Friedman PJ Leong L . The rivermead perceptual assessment battery in acute stroke. Br J Occup Ther 1992 Jun; 55: 233–237.

[15] Crawford JR Garthwaite PH . On the “optimal” size for normative samples in neuropsychology: capturing the uncertainty when normative data are used to quantify the standing of a neuropsychological test score. Child Neuropsychol 2008 Feb 28; 14: 99–117. 18306075 10.1080/09297040801894709

[16] Vancleef K Demeyere N . Oxford Visual Perception Screen Manual v2.1 [Internet]. 2023 [cited 2024 Dec 16]. Available from: https://oxvps.webspace.durham.ac.uk/wp-content/uploads/sites/370/2024/07/OxVPS-Manual-v2.1-01042024-double-sided.pdf

[17] Logarithmic Near Visual Acuit Chart 2000 ‘New ETDRS’ [Internet]. Precision Vision; 2023 [cited 2024 Jan 8]. Available from: https://www.sussex-vision.co.uk/distance-tests/Near-Vision-Tests/logmar-double-sided-near-vision-card

[18] Mimouni M Shamir RR Cohen A , et al. A comparison of different scoring terminations rules for visual acuity testing: from a computer simulation to a clinical study. Curr Eye Res 2019 Jul 3; 44: 790–795. 30829080 10.1080/02713683.2019.1589524

[19] Healthcare Quality Improvement Partnership. Sentinel Stroke National Audit Programme Clinical results 22/23 [Internet]. 2023 [cited 2024 Jan 19]. Available from: https://www.data.gov.uk/dataset/631f329d-069e-4565-9055-b793e2aa8492/sentinel-stroke-national-audit-programme-clinical-results-22-23

[20] Gittings NS Fozard JL . Age related changes in visual acuity. Exp Gerontol 1986 Jan 1; 21: 423–433. 3493168 10.1016/0531-5565(86)90047-1

[21] Erdinest N London N Lavy I , et al. Vision through healthy aging eyes. Vision 2021 Dec; 5: 46. 34698313 10.3390/vision5040046 PMC8544709

[22] Cooke DM McKenna K Fleming J , et al. Construct and ecological validity of the occupational therapy adult perceptual screening test (OT-APST). Scand J Occup Ther 2006 Jan; 13: 49–61. 16615415 10.1080/11038120500363014

[23] Cooke DM McKenna K Fleming J , et al. The reliability of the occupational therapy adult perceptual screening test (OT-APST). Br J Occup Ther 2005 Nov; 68: 509–517.

[24] Jesshope HJ Clark MS Smith DS . The rivermead perceptual assessment battery: its application to stroke patients and relationship with function. Clin Rehabil 1991 May; 5: 115–122.

[25] Office for National Statistics (ONS). Census 2021 data [Internet]. 2023 [cited 2023 Jul 27]. Available from: https://www.ons.gov.uk/census

[26] Lincoln NB Clarke D . The performance of normal elderly people on the rivermead perceptual assessment battery. Br J Occup Ther 1987 May; 50: 156–157.

[27] Cooke DM McKenna K Fleming J , et al. Australian Normative data for the occupational therapy adult perceptual screening test. Aust Occup Ther J 2006; 53: 325–336.

[28] Rivera Mindt M Byrd D Saez P , et al. Increasing culturally competent neuropsychological services for ethnic minority populations: a call to action. Clin Neuropsychol 2010 Apr 1; 24: 429–453. 20373222 10.1080/13854040903058960 PMC2909768

[29] Berry J Wallace KL Shores EA . The Chinese Australian neuropsychological normative study sample performance on Western and Chinese norms: caveats for cross-cultural neuropsychology. Aust Psychol 2019 Apr 1; 54: 90–101.

[30] Martinez GS Campbell AJ Reinken J , et al. Prevalence of ocular disease in a population study of subjects 65 years old and older. Am J Ophthalmol 1982 Aug 1; 94: 181–189. 7114140 10.1016/0002-9394(82)90073-3

[31] Webb SS Moore MJ Yamshchikova A , et al. Validation of an automated scoring program for a digital complex figure copy task within healthy aging and stroke. Neuropsychology 2021 Nov; 35: 847–862. 34618514 10.1037/neu0000748

